# Factors influencing patient delay in individuals with obstructive sleep apnoea: a study based on an integrated model

**DOI:** 10.1080/07853890.2022.2132417

**Published:** 2022-10-19

**Authors:** Hui Zhang, Chunguang Liang, Xin Zhang, Haitao Yu, Xiangru Yan, Liying Wang, Tong Tong, Huiying Zhang, Hongliang Dai, Huijuan Tong

**Affiliations:** a School of Nursing, Jinzhou Medical University, Jinzhou, PR China; b Department of Respiratory Medicine, Zibo Central Hospital, Zibo, PR China; c Department of Otolaryngology Head & Neck Surgery, First Affiliated Hospital of Jinzhou Medical University, Jinzhou, PR China; d Department of Nursing, Shenyang Medical College, Shenyang, PR China

**Keywords:** Health ecological model, theory of planned behaviour, sleep disorder, patient delay

## Abstract

**Background:**

Obstructive sleep apnoea (OSA) is the most common sleep-related breathing disorder, yet it remains undiagnosed in a large proportion of adults.

**Objective:**

This study aims to investigate the status of patient delay and provider delay in OSA patients and examine related factors affecting patient delay in OSA individuals in China.

**Methods:**

A cross-sectional design was conducted on a sample of 309 OSA patients (aged from 18 to 76, median age of 47 years, 84.8% male) in Northeast China. Participants were required to complete the sociodemographic questionnaire, the symptom characteristics questionnaire, the help-seeking attitude scale (HSAS), the social support rating scale and the chronic disease self-efficacy scales (CDSES) to test the hypothesis. Binary logistic regression analysis was conducted to explore factors that account for the patient delay.

**Results:**

The median patient delay among OAS patients in this study was 22 months, the median provider delay was one month, and the median total delay was 26 months. As shown by multivariate analysis results, patients who have snored for over 6 years (OR = 3.377, 95%CI: 1.175–9.702) were more likely to experience prolonged patient delays. Per capita monthly family income above 3000 RMB (OR = 0.172, 95%CI: 0.052–0.571), taking up residence in cities or towns (OR = 0.484, 95%CI: 0.248–0.946), higher self-recognition of the disease (OR = 0.793, 95%CI: 0.647–0.972), higher objective support (OR = 0.825, 95%CI: 0.739–0.921) and stronger self-efficacy (OR = 0.674, 95%CI: 0.525–0.867) were significantly associated with shorter patient delays.

**Conclusion:**

Patient delay is common in Chinese OSA patients. The upstream factors affecting the patient delay in individuals with OSA include income, place of residence, and objective support; midstream factors include self-recognition of the disease and self-efficacy; downstream factors include years of snoring.

**KEY MESSAGES**
- Despite being a high-prevalence disease, many obstructive sleep apnoea (OSA) patients are not clearly diagnosed and treated.
- The factors affecting the delay in seeking medical treatment in individuals with OSA included income, place of residence, objective support, self-recognition of the disease, self-efficacy and years of snoring.
- Investigations into OSA patients’ care-seeking behaviours can better reflect the secondary prevention of OSA, and it is crucial to pay attention to the delayed phase of patients.

## Introduction

Obstructive sleep apnoea (OSA) is a highly prevalent sleep disorder where patients experience during sleep the narrowing or blockage of their upper airway and muscle relaxation, which causes hypoventilation or apnoea and results in decreased oxygen saturation and microarousals [1]. The major symptoms of OSA include nocturnal snoring, breathing-related arousals, wheezing, daytime sleepiness, memory impairment and fatigue [2]. OSA not only affects multiple body systems and organs but also increases the risk of cardiovascular disease, diabetes, depression and all-cause mortality and improves the likelihood of road traffic or workplace accidents if left undiagnosed or untreated [3,4]. OSA has become a public health burden worldwide [5]. Studies have shown that OSA still remains massively underdiagnosed [6,7]. Many people with OSA do not seek medical attention after the onset of symptoms, and by the time they do, their disease has progressed to moderate and severe stages and may bring about a wide range of health consequences [6].

Compared with disease research, investigations into patients’ care-seeking behaviours can better reflect the secondary prevention of the disease. Care-seeking delays can be roughly divided into patient delay, provider delay and total delay [8]. Patient delay refers to the interval between symptom discovery and the first time the patients seek medical help. Most studies have always taken three months as the defining standard of the patient’s delay. If ≥3 months, the patient can be considered to have patient delayed; provider delay refers to the time from the patient’s first visit to diagnosis and treatment; the sum of patient delay and provider delay is the total delay. As proved by many studies, compared with provider delays, the time and health effects of patient delays are often longer and more profound, and patient factors have been highlighted as key contributors to total delays [9–11].

A variety of theories and models have been applied to analyse patient delay in health care-seeking behaviours, including psychological theories such as Leventhal’s theory of fear and danger control [12], Ajzen’s theory of planned behaviour (TPB) [13], and Andersen’s theory of total patient delay [14] and social theories such as the health belief model [15], the health ecology model [16], and Moodley’s treatment pathway model [17]. Previous studies have shown that patient delay is highly associated with socio-demographic factors, family support, self-cognitive, self-efficacy, health resilience and disease symptom characteristics [18–20].

Despite numerous investigations into the determinants of patient care-seeking, few studies have examined the impacts of multiple risk factors or protective factors at multiple levels. The health ecological model (HEM), a highly recognized model for examining the impacts of various factors at different levels, could link individual and social behaviours with environmental determinants [21]. HEM consists of five layers, with the core layer corresponding to the innate personality traits, including gender, age and susceptibility; The second layer comprises behavioural characteristics; the third layer corresponds to interpersonal network of society, family and community; the fourth layer corresponds to living and working conditions, including occupational factors, place of residence, economic status; and the fifth layer corresponds to the social, economic, cultural and other relevant policies both at local and national levels [22]. The upstream factors include the third, fourth and fifth layers, the midstream factors are the second layer, and the downstream factors are the core layer [23]. The HEM model has been used to investigate various types of behaviours, for example, in predicting and explaining eating behaviour [24] and exercise behaviours [25]. The TPB proposed by Ajzen in 1985 assumes that attitude towards behaviour, subjective norms and perceived behaviour control indirectly influence a person’s actual behaviour through behavioural intentions [26]. Behavioural intention and perceived behaviour control are directly associated with actual behaviour [26]. TPB theory has good predictive and explanatory power in many studies on patients’ health behaviours [27,28].

Both TPB theory and HEM can predict health care-seeking behaviours. We can see that there is an overlapping between the subjective norms in TPB theory and the connotations of upstream factors in HEM, and between perceived behavioural control and attitudes in TPB theory and the connotations of midstream factors in HEM. It should be noted that TPB theory and HEM have similarities as well as differences. This study adopts a combined model which integrates these two theories so as to achieve a higher degree of explanation in studies that explain and predict health care-seeking behaviours. The aim of this study is to investigate the status of patient delays and provider delays in OSA patients and to identify related factors that influence care-seeking behaviour on multiple levels. Thereby raising healthcare workers’ awareness of care-seeking delays among OSA patients and facilitating a comprehensive understanding of why patients delay seeking care for OSA.

## Methods

### Theoretical framework

**Figure 1.**
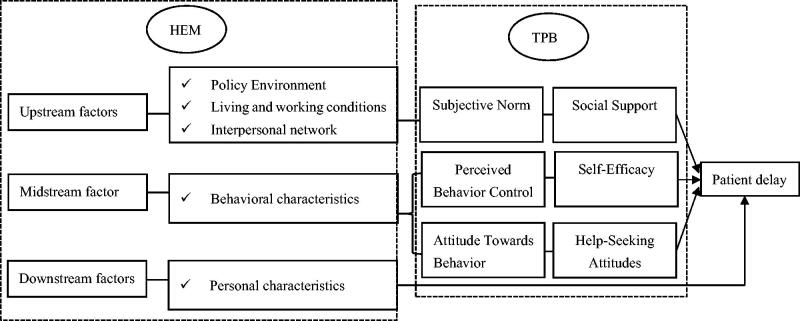
Hypothesised integrative model of patient delay among individuals with obstructive sleep apnoea. HEM: the health ecological model; TPB: the theory of planned behaviour.

The theoretical framework of this study (as shown in Figure 1) is derived from HEM and TPB theories. This study combined them to construct and integrate a theoretical framework with upstream, midstream and downstream factors that influence patients’ medical care seeking behaviour as entry points, and the variables included were sociodemographic characteristics, disease characteristics and psychosocial characteristics. Behavioural intention was not measured in this study because the recruited participants had already visited a physician when they participated in the survey.

All components of these two theories are given operational definition. In our study, the behavioural variable in TPB was operationally defined as ‘patient delay’, which refers to the time interval between the onset of symptoms and the patient’s first visit to a health care facility. Attitude towards behaviour was operationalized as ‘patient’s help-seeking attitudes of OSA’. The subjective norm was operationally defined as ‘social support’, which refers to the influence exerted by others on the OSA patient’s medical behaviour. Perceived behavioural control was operationalized as ‘self-efficacy’, which refers to OSA patients’ confidence in coping with the disease.

### Study design and participants

A questionnaire-based cross-sectional survey was conducted in this study. Patients who attended the sleep monitoring centres at the First and the Third Affiliated Hospitals of Jinzhou Medical University from September 2020 to October 2021 and were diagnosed with OSA were included in this study. The following criteria were included when selecting participants: (a) age ≥18 years; (b) diagnosed OSA according to standard criteria [29]; (c) able to recall the time of the initial discovery of abnormal symptoms; (d) willing to participate in the study. The exclusion criteria were: (a) Patients who refused to provide written informed consent; (b) unstable health conditions, such as congestive heart failure, malignancy, or end-stage renal failure; (c) those with hearing, speech, and other communication impairments; (d) those with a history of depression, dementia or those currently taking psychotropic medication.

### Procedure

The procedure of this study involves a face-to-face questionnaire interview and collection of disease information. The survey group was composed of two postgraduates and two nurses, and the survey was conducted in a separate and quiet room. The investigators were uniformly trained prior to the survey and all the patients were informed of the purpose and significance of the survey. All participants were kept anonymous. Since the data were collected by a paper-based questionnaire, for those unable to complete the questionnaire due to visual impairment, the investigators read and explained the items to them without any hints of personal preference and asked them to answer the questions verbally.

### Ethics approval

The research procedures complied with the ethical standards of the 1964 Helsinki declaration and its later amendments. The study protocol was approved by the Ethics Committee of the Jinzhou Medical University (approval number: LLSC2020008).

### Instruments

The research instruments used in this study consist of five parts, including demographic characteristics and disease symptom characteristics that are specific to this study, the help-seeking attitude scale (HSAS), the Social Support Rating Scale (SSRS) and the chronic disease self-efficacy scale (CDSES).

The demographic characteristics questionnaire was designed by our research team based on the HEM theory. Downstream factors include sex, age and comorbid chronic diseases; midstream factors include smoking behaviour, alcohol drinking behaviour and regular physical examination status; upstream factors include marital status, employment status, place of residence, education background, per capita family monthly income and the impact of COVID-19 on patients seeking medical treatment.

Disease symptom characteristics items were determined according to the results of a comprehensive literature review and clinical experts’ suggestions. Relevant items in this section were filled in by the investigators. In the first step, sleep breathing parameters, the patient’s body mass index (BMI), and the date of OSA diagnosis were obtained from the patient’s sleep monitoring report. Next, patients were asked to report their medical visits, including (a) the first symptoms of OSA (i.e. sleep or body abnormal symptoms occurring to their sleep or body that they first discovered, such as snoring, suffocating, awakening during sleep, morning headache or dry mouth, daytime sleepiness, hypertension, which could be multiple choices); (b) the time of the first onset of the above symptoms; (c) time of their first visit to doctors; (d) years of snoring. The investigators then calculated the range of patient delay, the interval from the first symptom or sign to the first medical treatment, and the range of provider delay, the interval from the first medical visit to OSA diagnosis. The interviewer used closed questions to collect data about the onset of symptoms and the date of their first healthcare attendance. When doing the survey, the researchers adopted a calendar anchoring technique to facilitate accurate date recall if the OSA patient had difficulty to do so [30]. In addition, there is evidence that partners or family members of OSA patients are helpful for patients’ recalling of the critical information [31]. Thus, patients were allowed to ask their spouse, partner, and family members for help during the survey.

Patients’ help-seeking attitudes were measured by a scale that was used to assess incontinent women’s help-seeking attitudes developed by Sun Tao [32]. For this study, ‘urine leakage’ was replaced with ‘snoring, sleep apnoea symptoms’. The scale comprises 12 items that fall into three dimensions, namely self-recognition, emotional experience and help-seeking intention. The scale scored using a 5-point Likert format (5 = ‘extremely disagree’, 4 = ‘disagree’, 3 = ‘not sure’, 2 = ‘agree’, 1 = ‘extremely agree’). Among the 12 items, Items 3 and 6 were positively scored questions. The total score indicates the patient’s help-seeking attitude towards OSA diagnosis and treatment, with a lower score indicating a more negative attitude towards seeking medical treatment. In 2018, Shi and Yang adopted the scale in a research investigation of haemorrhoids patients’ delay in seeking medical treatment. They tested the reliability of the scale and found that the overall Cronbach’s α of the scale was 0.716, suggesting that the scale had good reliability [33]. In this study, the Cronbach’s α of the scale was 0.675.

The Social Support Rating Scale developed by Xiao Shuiyuan in 1986 was adopted to measure social support. The ten-item scale consists of three dimensions, including objective support, subjective support and the utilization of support [34]. The sum of the scores obtained on the ten items is the total score. The scale has good reliability and validity in that the consistency of each item is between 0.890 and 0.896 and that the test-retest reliability is 0.921. In this study, the Cronbach’s α of this scale was 0.769.

The CDSES was developed by Lorig et al. in 1996 to evaluate the self-efficacy of patients with chronic diseases [35]. The six-item scale which incorporates different domains is suitable for measuring participants’ confidence levels in areas such as controlling symptoms, functioning role, emotional functioning and communicating with the physician. Each item was rated on a visual simulation scale from 1 to 10, where 1 point means ‘no confidence at all’ and 10 points means ‘absolute confidence’. The average of the scores on the six items is the total score, ranging from 1 to 10 points, with higher scores indicating higher self-efficacy. The Chinese version of this scale has been widely used and proved to have good reliability and validity [36]. As tested on the OSA patients in this study, the Cronbach’s α of the scale was 0.938.

### Sleep monitoring

All subjects were monitored by sleep polysomnography (N7000) from EMBLA company or portable sleep monitors (X100) for more than seven hours per night. The obtained data were interpreted by investigators according to the multi-channel sleep breathing interpretation manual formulated by the American Academy of Sleep Medicine (AASM) [29], analysed by a professional sleep technician and verified by a sleep physician. The severity of OSA is defined by the apnoea-hypopnea index (AHI), is indicated by the average number of abnormal respiratory events per hour of sleep. According to the criteria of the AASM, the severity of OSA can be classified as mild, moderate or severe levels, corresponding to AHI from 5 to 15, 15 to 30 or higher than 30 events per hour of sleep, respectively. The lowest arterial oxygen saturation (LSaO_2_) was used to assess the severity of hypoxaemia: 85–90%, 80–85% or <80%, indicating mild, moderate or severe hypoxaemia, respectively [29]. The BMI values of the patients were also obtained from the monitoring report. Participants were categorized as underweight (<18.5 kg/m^2^), normal weight (18.5–23.9 kg/m^2^), overweight (24–27.9 kg/m^2^) and obese (≥28 kg/m^2^) according to the Chinese criteria of weight for adults [37].

### Statistical analysis

Data analysis was performed using SPSS version 25.0 (SPSS Inc., Chicago, IL) and data were summarized by standard descriptive statistics. Kolmogorov–Smirnov normality tests were conducted to examine the distribution of the continuous variables. In all the subjects, the sample size of non-delayers was much smaller than that of delayers and no comparability was observed between the two groups. The delayers were dichotomized using the 50th percentile as the cut-off value, thus the cut-off points of <24 months and ≥24 months were chosen to distinguish between short delayers and long delayers. The t-test or ANOVA was used for the measurement data that obeyed the normal distribution, and the Mann–Whitney U test was used if it conformed to the non-normal distribution. A chi-square test was performed to compare the demographic statistics of the two groups. All independent variables were checked for collinearity. Patient delay (short or long delays) served as the dependent variable, and factors significantly related to patient delay were then entered into a Binary logistic regression analysis to test their significance. The results were expressed as odds ratio (OR) and 95% confidence interval (95% CI). A *p* value <0.05 (two-tailed) was considered statistically significant for all studies.

## Results

### Descriptive statistics

A total of 334 questionnaires were distributed and all of them were collected in this study, and 25 invalid questionnaires (with missing information or incomplete questionnaires) were excluded. A total of 309 (92.51%) questionnaires from OSA patients were finally included in the final analysis. In our study, most subjects were male (84.8%, *n* = 262). Among the 309 patients, their ages ranged from 18 to 76 years old, with the median age of 47 years old, and 69.5% of the patients were older than 40 years old. Seventy-seven patients (24.9%) thought that the current epidemic normalized prevention and control affected their decision to seek medical treatment. Among these patients, 16 patients (20.8%) believed that inconvenient transportation hindered their medical care-seeking behaviour; 31 patients (40.3%) held that the epidemic made the medical treatment more complicated; 24 patients (31.1%) worried about the risk of infection while seeking medical treatment, and the other 6 patients (7.8%) believed that other reasons caused by the epidemic had affected their medical treatment decision.

Among the subjects in this study, the severity of OSA was predominantly moderate and severe, accounting for 88.1% of the cases. AHI ranged from 6.0 to 112.0, with the median AHI of 43.1; LSaO2 ranged from 90.0 to 50.0, with the median LSaO2 of 80.0; BMI was the highest at 43.0 and the lowest at 18.1 with the median BMI of 28.1. Other detailed information of the subjects is shown in Table 1.

**Table 1.** Sociodemographic information and disease characteristics s of the subjects (*n* = 309).

Variables	Options	*N*	Percentage
Gender	Male	262	84.8
	Female	47	15.2
Age (years)	<40	94	30.4
	40∼	175	56.7
	60∼	40	12.9
Marital status	Never married	30	9.7
	Married/cohabitated	253	81.9
	Divorced/widowed	26	8.4
Employment status	In-service	184	59.5
	Retired	45	14.6
	Unemployed	80	25.9
Education background	Primary school	65	21.0
	Middle school	89	28.8
	High school	103	33.3
	≥Graduate	52	16.8
Per capita monthly family income (RMB)	<1500	46	14.9
	1500∼	111	35.9
	3000∼	93	30.1
	4500∼	59	19.1
Place of residence	Rural area	143	46.3
	City or town	166	53.7
Smoking behaviour	No smoking	130	42.1
	Occasional smoking	84	27.2
	Regular smoking	95	30.7
Alcohol drinking behaviour	No alcohol drinking	140	45.3
	Occasional drinking	85	27.5
	Regular drinking	84	27.2
Impact of COVID-19	No impact	232	75.1
	having impact	77	24.9
Regular physical examination status	Yes	169	54.7
	No	140	45.3
Comorbid chronic diseases	One or more	205	66.3
	No	104	33.7
AHI	Mild	37	12.0
	Moderate	70	22.7
	Severe	202	65.4
LSaO_2_	Mild	81	26.2
	Moderate	75	24.3
	Severe	153	49.5
Years of snoring	<3	64	20.7
	3∼	53	17.2
	6∼	47	15.2
	10∼	145	46.9
BMI	Underweight	1	0.3
	Normal weight	25	7.8
	Overweight	129	41.7
	Obese	155	50.2

AHI: apnoea-hypopnea index; LSaO_2_: lowest arterial oxygen saturation; BMI: body mass index

Many OSA patients had two or more first symptoms (see Table 2), among which the most common ones were snoring and sleep apnoea. For some patients (about 27.18%) with other diseases such as hypertension, diabetes, cerebral vascular disease, arrhythmia, coronary heart disease, gastroesophageal reflux disease, the driving force for their first visit to doctors was not OSA-related symptoms, instead they were referred from other departments.

**Table 2.** Symptom type of the participants (*n* = 309).

Symptoms	*N*	Pct of responses	Pet of cases
Soring	227	33.58	73.46
Waking up, suffocation during sleep	216	31.95	69.90
Dizziness, headache, dry mouth	53	7.84	17.15
Daytime sleepiness, fatigue	70	10.36	22.65
Cardiovascular disease, cerebrovascular disease, diabetes, hypertension, etc.	84	12.43	27.18
Other clinical symptoms or diseases^a^	26	3.84	8.50
Total	676	100.00	218.77

The pct of responses in the table was calculated as the ratio of the occurrence of a certain symptom to the total number of occurrences of all symptoms; the pct of cases was calculated as the ratio of patients with this symptom to the total subjects; ^a^other clinical symptoms or diseases included cough, reflux disease, nightmares, etc.

### Status of patient delay, provider delay and total delay in OSA patients

The patients were grouped into delayed and non-delayed groups with the criterion of a 3-month time point. The survey results showed that 284 OSA patients had medical treatment-seeking delay and the delay rate was 91.9%. Descriptive statistics are shown in Table 3.

**Table 3.** Descriptive statistics of patient delay (*n* = 309).

	Maximum value	Minimum value	Median	IQR
Total	456.0	0.5	22.0	37.0
Delay group	456.0	3.0	24.0	37.5
Non-delay group	2.0	0.5	2.0	0.5

The time unit is the month.

The median provider delay was one month (ranging from 0.5 to 28 months). The segmentation criterion of ‘<1 month, 1∼, 3∼, 6∼, 9∼, 12∼’ was used to describe the delay in OSA diagnosis. Descriptive statistics are shown in Table 4. About 70.2% of the patients were diagnosed within 3 months after their first visit, and the total delay from symptom onset to OSA diagnosis among the OSA patients was 26 months (IQR = 37).

**Table 4.** Descriptive statistics of provider delay (*n* = 309).

	<1	1∼	3∼	6∼	9∼	12∼
*N*	98	119	24	22	14	32
Percentage	31.7	38.5	7.8	7.1	4.5	10.4

The time unit is the month.

### Factors affecting patient delay among OSA patients

According to the delay time, 284 delayers were divided into short and long delay groups. Table 5 shows the univariate analysis of factors affecting the patient delay.

**Table 5.** The univariate analysis of factors affecting the patient delay.

	*N* (%) or median (IQR)			
Variables	Short-delay (*n* = 135)	Long-delay (*n* = 149)	χ^2^/Z value	*p*
Upstream factors				
Marital status			1.736	0.420
Never married	15 (11.1)	11 (7.4)		
Married/Cohabitated	111 (82.2)	124 (83.2)		
Divorced/Widowed	9 (6.7)	14 (9.4)		
Employment status			5.122	0.077
In-service	89 (65.9)	79 (53.0)		
Retired	15 (11.1)	26 (17.4)		
Unemployed	31 (23.0)	44 (29.5)		
Education background			31.777	**0.000**
Primary school	15 (11.1)	46 (30.9)		
Middle school	33 (24.2)	51 (34.2)		
High school	54 (40.0)	41 (27.5)		
≥Graduate	33 (24.4)	11 (7.4)		
Per capita monthly family income (RMB)			33.214	**0.000**
<1500	8 (5.9)	34 (22.8)		
1500∼	39 (28.9)	65 (43.6)		
3000∼	53 (39.3)	34 (22.8)		
4500∼	35 (25.9)	16 (10.7)		
Place of residence			28.181	**0.000**
Rural area	40 (29.6)	91 (61.1)		
City or town	95 (70.4)	58 (38.9)		
Impact of COVID-19			3.980	**0.046**
No impact	95 (70.4)	120 (80.5)		
Having impact	40 (29.6)	29 (19.5)		
Objective support	8.0 (5.0)	6.0 (4.0)	−3.915	**0.000**
Subjective support	18.0 (4.0)	16.0 (5.0)	−3.465	**0.001**
Utilization of support	7.0 (3.0)	6.0 (3.0)	−1.706	0.088
Midstream factors				
Smoking behaviour			4.542	0.103
No smoking	56 (41.5)	62 (41.6)		
Occasional smoking	43 (31.9)	33 (22.1)		
Regular smoking	36 (26.7)	54 (36.2)		
Alcohol drinking behaviour			6.435	**0.040**
No drinking	63 (46.7)	64 (43.0)		
Occasional drinking	43 (31.9)	34 (22.8)		
Regular drinking	29 (21.5)	51 (34.2)		
Regular physical examination			7.915	**0.005**
Yes	85 (63.0)	69 (46.3)		
No	50 (37.0)	80 (53.7)		
Self-efficacy	7.5 (1.7)	6.5 (2.0)	−6.430	**0.000**
Self-recognition	9.0 (2.0)	9.0 (8.0)	−3.668	**0.000**
Emotional experience	18.0 (8.0)	17.0 (6.5)	−1.275	0.202
Help-seeking intention	9.0 (2.0)	9.0 (2.0)	−1.863	0.062
Downstream factors				
Gender			0.113	0.736
Male	115 (85.2)	129 (86.6)		
Female	20 (14.8)	20 (13.4)		
Age (years)			15.152	**0.001**
<40	53 (39.3)	33 (22.1)		
40∼	73 (54.1)	88 (59.1)		
60∼	9 (6.7)	28 (18.8)		
Comorbid chronic diseases			13.746	**0.000**
One or more	30 (22.2)	64 (43.0)		
No	105 (77.8)	85 (57.0)		
AHI			0.409	0.815
Mild	17 (12.6)	18 (12.1)		
Moderate	32 (23.7)	31 (20.8)		
Severe	86 (63.7)	100 (67.1)		
LSaO_2_			1.134	0.567
Mild	32 (23.7)	40 (26.8)		
Moderate	37 (27.4)	33 (22.1)		
Severe	66 (48.9)	76 (51.0)		
Years of snoring			35.143	**0.000**
<3	43 (31.9)	15 (10.1)		
3∼	29 (21.5)	15 (10.1)		
6∼	17 (12.6)	26 (17.4)		
10∼	46 (34.1)	93 (62.4)		
BMI			3.177	0.204
Underweight	1 (0.7)	0 (0.0)		
Normal	9 (6.7)	9 (9.0)		
Overweight	65 (48.1)	58 (38.9)		
Obese	60 (44.4)	82 (55.0)		

AHI: apnoea-hypopnea index; LSaO_2_: lowest arterial oxygen saturation; BMI: body mass index

Bold values correspond to statistically significant correlations (*p* < 0.05).

**Table 6.** Logistic regression analysis of factors associated with patient delay.

Variable	Comparison group	Reference group	*β*	S.E.	Waldχ^2^	*p*	OR	95%CI
Age (year)	40∼	<40	−0.221	0.408	0.294	0.587	0.802	0.360 − 1.783
	60∼		0.623	0.660	0.893	0.345	1.865	0.512 − 6.794
Education background	Middle school	Primary school	−0.043	0.468	0.009	0.926	0.958	0.383 − 2.396
	High school		−0.002	0.511	0.000	0.996	0.998	0.366 − 2.718
	≥Graduate		0.035	0.746	0.002	0.962	1.036	0.240 − 4.467
Income (RMB)	1500∼	<1500	−0.861	0.535	2.586	0.108	0.423	0.148 − 1.207
	3000∼		−1.758	0.611	8.272	**0.004**	0.172	0.052 − 0.571
	4500∼		−1.464	0.721	4.118	**0.042**	0.231	0.056 − 0.951
Place of residence	City or town	Rural area	−0.725	0.342	4.511	**0.034**	0.484	0.248 − 0.946
Drinking behaviour	Occasional	No drinking	−0.358	0.380	0.887	0.346	0.699	0.332 − 1.472
	Regular drinking		0.034	0.388	0.008	0.931	1.034	0.484 − 2.212
Impact of COVID-19	Having impact	No impact	0.091	0.391	0.054	0.816	1.095	0.509 − 2.358
Chronic diseases	One or more	No	−0.081	0.368	0.049	0.825	0.922	0.448 − 1.898
Physical examination	No	Yes	−0.134	0.338	0.158	0.691	0.874	0.451 − 1.696
Years of snoring	3∼	<3	0.356	0.532	0.448	0.503	1.428	0.503 − 4.049
	6∼		1.217	0.538	5.108	**0.024**	3.377	1.175 − 9.702
	10∼		1.270	0.496	6.540	**0.011**	3.559	1.345 − 9.418
Self-recognition			−0.232	0.104	4.974	**0.026**	0.793	0.647 − 0.972
Objective support			−0.192	0.056	11.726	**0.001**	0.825	0.739 − 0.921
Subjective support			0.009	0.046	0.035	0.851	1.009	0.922 − 1.103
Self-efficacy			−0.394	0.128	9.464	**0.002**	0.674	0.525 − 0.867
Constant			6.853	1.558	19.339	0.000		

β: standardized Beta coefficient; S.E: standard error; OR: odds ratio; CI: confidence interval

Bold values correspond to statistically significant correlations (*p* < 0.05).

Binary logistic regression analysis was performed with different degrees of patient delay as dependent variables (short delay = 0, long delay = 1) and statistically significant variables in the univariate analysis as independent variables. After the collinearity test, the variance inflation factor (VIF) is between 1.088 and 1.191, which is far below the critical standard of five, so no collinearity problem exists. The results are shown in Table 6. The results showed that the per capita monthly income of the family, the place of residence, years of snoring, self-recognition of the disease, objective support and self-efficacy were independent factors accounting for the delay in seeking medical treatment among OSA patients.

## Discussion

Many patients with OSA usually do not seek prompt medical attention after the first symptoms, resulting in a high prevalence of undiagnosed OSA. An integrated model is adopted in this study to investigate the current status of patient delay and provider delay among 309 OSA patients in Jinzhou, China. It was found that delays in seeking medical treatment among OSA patients are affected by multiple factors at multiple levels, among which the upstream factors include income, place of residence and objective support; midstream factors are the self-recognition of the disease and self-efficacy of patients and downstream factors include years of snoring.

The results showed that the median patient delay of the patients was 22 months, and the rate of patient delay (length of delay ≥3 months) was 91.9%. Franck surveyed 97 OSA patients and found that the average time between the onset of the main symptom of OSA and referral to a sleep centre was 87.5 months [9]; Henry investigated the time lapse from the first awareness of a problem to actually seeking medical help in OSA patients, the average time lapse reported by patients was 4.8 ± 5.8 years [31]. These results suggested a high rate of delay in seeking medical treatment among OSA patients. However, very few results have been reported in China on the duration of delayed access to medical care among OSA patients. The proportion of moderate and severe OSA patients in this study takes up 88.1% of all the patients. Early diagnosis is critical to slowing the progression of OSA disease, reducing target organ damage and mitigating the risk of death [38].

This survey found that most patients with OSA (70.2%) were diagnosed within 3 months of their first visit, but 32 patients (10.4%) still had more than 12 months between their first visit and the diagnosis of OSA. The provider delay for OSA is much shorter compared to patient delay. However, compared to other chronic diseases like diabetes, hypertension and chronic obstructive pulmonary disease, etc., there is still a lack of public awareness and more effective diagnosis of OSA [39]. Also, OSA has non-specific signs and symptoms, which may be a cause of provider delay. In this study, 27.18% of the subjects were referred from other departments. Lucas’ study found a high prevalence of OSA in cardiovascular-related disease departments [40]; In addition, many doctors currently lack the awareness or ability to recognize and diagnose OSA in a timely manner [41]. A study by Thornton et al. showed that the average waiting time for patients with sleep disorders to be referred to a sleep centre for PSG monitoring was 88 d. Many hospitals lack sleep diagnostic equipment and limited sleep medicine resources are unable to meet the needs of all patients [42].

Upstream factors include interpersonal, work, life and social factors. Per capita monthly household income indicates the patient’s economic situation and patients with higher per capita monthly household income are less likely to postpone their medical treatment. A study by Majeed et al. showed that economic factors hinder people’s use of medical services after illness [43]. Annual income affects the medical treatment behaviour of the floating population in that high-income individuals are more inclined to choose better medical institutions for medical treatment [44]. Tarasiuk’s study found that 72% of low-income patients refused to use CPAP devices. However, when the devices were subsidized, the acceptance rate increased by 43% [45]. The above-mentioned results prove that economic factors greatly influence the healthcare decisions of OSA patients.

This study also found that OSA patients living in rural areas experienced longer delays in seeking medical care. With increases in the distance from place of residence to health care facility comes the higher likelihood that residents will delay access to medical care and select some self-care approaches, a phenomenon associated with the lower accessibility to medical treatment services in rural areas [46]. Allen et al. reached the same conclusion that the farther the residence is from the sleep monitoring centre, the less likely a patient is to seek medical attention [47]. Many patients living in rural areas and towns tend to seek medical treatment in the nearest primary hospitals. However, these primary hospitals are faced with limited capacity of sleep disorder diagnosis and treatment, and misdiagnosis and missed diagnosis of OSA may occur in these hospitals [48], which then leads to the more prominent problem of delayed medical treatment for such OSA patients.

Objective support refers to realistic and visible support, including material support and group relationship support such as from family, friends and marriage partner [34]. Zarhin’s study showed that family ties are helpful in symptom recognition and in facilitating medical consultation, so patients living alone are more likely to have delays in seeking medical attention than those living with family [49]. Family support is beneficial to the management and prognosis of individuals with OSA. Seixas et al. conducted sleep health training for black companions at high risk of OSA in a randomized control trial to provide more objective support for this group of people. The results showed that sleep health training can improve the compliance of high-risk black companions in sleep assessment and treatment [50].

In this study, the epidemic impact variable was included as an upstream factor that may affect the patient delay. The rapid and wide spread of COVID-19 has caused a worldwide rampage of neo-coronavirus pneumonia [51]. According to a systematic review, COVID-19 has seriously affected the diagnosis, treatment and management of patients with chronic diseases like OSA [52]. However, the multivariate analysis results in this study showed that the epidemic’s impact was not an independent factor accounting for the OSA patients’ delay in seeking medical treatment. Considering that there are various degrees of COVID-19 impact in different regions, this study only investigated OSA patients in Jinzhou, thus the observations in this study are not generalizable to other regions. Future research is recommended to carry out a retrospective study to compare patient delay in seeking medical treatment before and after the epidemic.

The midstream factors are factors related to individual behavioural characteristics. Self-recognition of disease refers to the individual’s understanding of the concept, aetiology and symptoms of the disease. Self-recognition of the disease (OR = 0.793, *p* < 0.05) as a protective factor for the occurrence of prolonged delay in seeking medical care in patients with OSA. To be specific, the stronger the self-recognition of the disease, the shorter the delay in seeking medical treatment. A previous study showed an average of 17.2 visits per person to primary care clinics from the first symptoms to OSA diagnosis, suggesting that patients fail to take advantage of many opportunities for OSA evaluation and diagnosis [53]. Raphael found that African American groups often regard OSA as insomnia or an age-related manifestation rather than a disease. Due to these misperceptions, this group is less likely to be evaluated and treated for OSA [54], and misjudgement of symptoms will significantly delay patients from seeking medical care. Many surveys have found that the public generally has low awareness of OSA and a low level of attention [55]. Therefore, it is urgent to make people aware of OSA through some feasible ways, including increasing OSA patients’ attention to symptoms, updating their perception of the disease and their way of coping with the disease, thus encouraging them to seek medical treatment on time. More efforts should be made to develop health care education about early detection of OSA to strengthen individuals’ health awareness, increase their knowledge of symptoms and promote their early consultation of the disease. Self-efficacy in this study refers to OSA patients’ confidence in taking on self-care behaviours and coping with related symptoms such as snoring and sleepiness. A study showed that self-efficacy in OSA patients affects compliance with CPAP therapy [56]. Our results demonstrated that self-efficacy also affects the delay in care-seeking behaviours among OSA patients. Similarly, previous studies have found that the higher the level of self-efficacy of patients with breast cancer, the more likely they are to adopt positive health behaviours and the less likely they are to have delayed medical care-seeking behaviour [57].

The downstream factors include factors related to patients’ innate personal characteristics. This study found that OSA patients who had snored for more than ten years had a 3.559-fold increased risk of delay in seeking medical treatment than those who had snored for less than 3 years. Snoring as the most common symptom of OSA patients is often one of the first symptoms that appear at an early stage. In this study, snoring was found to be one of the first symptoms among 73.46% of the patients. Numerous epidemiological surveys have demonstrated the prevalence of snoring among adolescents and adults [58,59]. The lack of awareness of OSA makes it difficult for potential OSA patients to recognize snoring as an informative indicator of OSA, thus contributing to OSA patients’ delays in seeking care. A survey showed that snoring is considered a stigma in the United States, as well as obesity, and that the first reaction of many OSA patients to apnoea is to simply deny the disease [60]. Studies have confirmed that the stigma associated with many diseases or related symptoms (e.g. AIDS, cancer and obesity) contributes to patients’ avoidance of medical attention [61,62]. The stigma that snoring brings to patients may be one reason that drives patients to avoid seeking medical care. What is more important, OSA as a chronic disease will gradually make patients accept and adapt to the pains it brings, and the more patients get used to the symptoms, the less likely they are to seek medical care because of their snoring.

### Limitations

Several limitations should be considered when interpreting the findings of this study. First, this study asked patients to recall early symptoms, and when these symptoms occurred, thus bias was inevitable because of the self-reporting nature of this investigation. Second, in this study, the patient delay might have been overestimated among those without OSA-specific symptoms (e.g. patients with only snoring symptom or cardiovascular symptom). Thirdly, the participants in this study were selected from two Grade III-A comprehensive hospitals in Liaoning Province, which may limit the generalizability of the research findings. Finally, this study mainly investigated the influencing factors accounting for patient delay, so future studies are suggested to explore the factors affecting provider delay.

## Conclusions

The aim of this study was to investigate the current situation of patient delay among OSA patients and to identify the factors accounting for patient delay. It is concluded that patient delays in OSA are highly prevalent in China. The total medical delay in OSA is more attributable to patient delays than to provider delays. Our study has been the first study that sets out to identify factors affecting the delays of medical care-seeking behaviour among OSA patients supported by a theoretical framework. Our study reaches the conclusion that income, place of residence, objective support, self-recognition of the disease, self-efficacy, and snoring years are factors accounting for OSA patients’ delay in seeking medical care. These identified factors should be considered when designing intervention programs for the early identification of high-risk individuals and the reduction of patient delay.

## Data Availability

The data that support the findings of this study are available from the corresponding author CL, upon reasonable request.
